# The Entity of Connshing Syndrome: Primary Aldosteronism with Autonomous Cortisol Secretion

**DOI:** 10.3390/diagnostics12112772

**Published:** 2022-11-13

**Authors:** Mara Carsote

**Affiliations:** Department of Endocrinology, Carol Davila University of Medicine and Pharmacy & C.I. Parhon National Institute of Endocrinology, 011683 Bucharest, Romania; carsote_m@hotmail.com

**Keywords:** Connshing syndrome, Conn syndrome, Cushing syndrome, aldosterone, cortisol, adrenal tumour, primary aldosteronism, adrenal vein, CYP11B1/B2, adrenalectomy

## Abstract

Connshing syndrome (CoSh) (adrenal-related synchronous aldosterone (A) and cortisol (C) excess) represents a distinct entity among PA (primary hyperaldosteronisms) named by W. Arlt et al. in 2017, but the condition has been studied for more than 4 decades. Within the last few years, this is one of the most dynamic topics in hormonally active adrenal lesions due to massive advances in steroids metabolomics, molecular genetics from CYP11B1/B2 immunostaining to genes constellations, as well as newly designated pathological categories according to the 2022 WHO classification. In gross, PA causes 4–10% of all high blood pressure (HBP) cases, and 20% of resistant HBP; subclinical Cushing syndrome (SCS) is identified in one-third of adrenal incidentalomas (AI), while CoSh accounts for 20–30% to 77% of PA subjects, depending on the tests used to confirm autonomous C secretion (ACS). The clinical picture overlaps with PA, hypercortisolemia being mild. ACS is suspected in PA if a more severe glucose and cardiovascular profile is identified, or there are larger tumours, ACS being an independent factor risk for kidney damage, and probably also for depression/anxiety and osteoporotic fractures. It seems that one-third of the PA-ACS group harbours mutations of C-related lines like *PRKACA* and *GNAS*. A novel approach means we should perform CYP11B2/CYP11B1 immunostaining; sometimes negative aldosteronoma for CYP11B1 is surrounded by micronodules or cell clusters with positive CYP11B1 to sustain the C excess. Pitfalls of hormonal assessments in CoSh include the index of suspicion (check for ACS in PA patients) and the interpretation of A/C ratio during adrenal venous sample. Laparoscopic adrenalectomy is the treatment of choice. Post-operative clinical remission rate is lower in CoSh than PA. The risk of clinically manifested adrenal insufficiency is low, but a synthetic ACTH stimulating testing might help to avoid unnecessary exposure to glucocorticoids therapy. Finally, postponing the choice of surgery may impair the outcome, having noted that long-term therapy with mineralocorticoids receptors antagonists might not act against excessive amounts of C. Awareness of CoSh improves management and overall prognosis.

## 1. Introduction

Connshing syndrome (adrenal-related over production of aldosterone and cortisol) represents a challenge in the heterogeneous and dynamic field of adrenal masses [1,2,3,4]. While both diseases, Conn syndrome and Cushing syndrome, are traditional endocrine disorders, this entity, named “Connshing” by W. Arlt et al., continues to capture a lot of attention especially within last years, and yet there is much still to understand about this entity [1]. In 2017, a team of scientists from the University of Birmingham studied the steroid metabolomics of patients with primary aldosteronism (PA) and identified a subgroup of them with glucocorticoid excess, the combination has been recognized as a distinct aetiology of secondary high blood pressure (HBP) [1,2]. However, this specific hormonal combination is actually identified in a multitude of historic and recent studies, but not under this specific nomenclature.

Conn syndrome represents the most frequent cause of secondary HBP (approximately 4% to 9–10% of patients with HBP, and one fifth of the subjects with resistant HBP, but it is frequently underdiagnosed). The condition goes beyond cardiovascular impacts like neurological and renal complications of long standing, uncontrolled HBP, abnormal electrolytes profile, and pro-inflammatory status, to being associated with an increased risk of excessive insulin release and insulin resistance complicated with diabetes mellitus, a higher rate of metabolic syndrome features, of osteoporotic fractures, and depression when compared to the general hypertensive population [5,6,7,8,9,10,11].

On the other hand, adrenal Cushing syndrome relates either to benign adenomas or bilateral adrenal lesions (bilateral macronodular or micronodular adrenal cortical diseases), or to adrenocortical carcinoma [12,13,14,15,16,17,18,19,20]. In terms of clinical expression, there may be an overt clinical presentation or a (traditionally) so-called subclinical Cushing syndrome, a term underlying autonomous cortisol secretion (ACS) or possible ACS [12]. The medical and social burden of clinically manifested Cushing syndrome and to a lesser extent of mild hypercortisolism—despite not having the global epidemiological impact of PA—is caused by multi-organ morbidity and mortality due to HBP, diabetes mellitus, thromboembolism, obesity, myopathy, osteoporosis, infections, depression and neurocognitive impairment, etc. [12,13,14,15,16,17,18,19,20,21]. Subclinical Cushing syndrome (which is the type of cortisol excess most associated with PA in Connshing syndrome) is generally identified in 5–50% (roughly, one-third) of adrenal incidentalomas, depending on endocrine tests used in order to confirm the cortisol over production [22]. A prevalence of subclinical Cushing syndrome up to 2% among individuals of 60 years and older or an even a higher ratio (10%) in adults with uncontrolled HBP and/or diabetes mellitus, or low-trauma or spontaneous (osteoporotic) fractures, is reported [23].

The purpose of this research is to highlight an update on adrenal lesions manifested with persistent production of both aldosterone (PA) and cortisol (ACS), namely Connshing syndrome. 

## 2. Methods

This is a narrative review on English literature concerning full length articles on PubMed with no time line restriction. The key words of research were “Connshing”, and different combinations of “cortisol” (alternatively, “hypercortisolemia”, “Cushing syndrome”) and “aldosterone” (alternatively, “primary aldosteronism”, “hyperaldosteronism” or “Conn syndrome”), as well as “adrenal tumour” and “adrenal incidentaloma”. We organized the review into several sections from epidemiological perspective and clinical presentation to hormonal, molecular and genetic evaluation, as well as disease management.

## 3. Approach of Connshing Syndrome

### 3.1. The Concept of Connshing Syndrome

Despite the fact that actually the combination of adrenal cortical—associated aldosterone with cortisol excess was first reported in 1979, and noting that doctor Jerome Conn described PA in 1955, followed by doctor Michael Lityński in 1956, the entity of Connshing syndrome has been the subject of growing evidence and received massive attention only recently due to advance in steroids metabolomics, immunohistochemistry and molecular genetics of adrenal glands, and, probably, a more extensive use of adrenal venous sampling (AVS) [12,20,24,25,26].

In the field of adrenal tumours, there has been also a terminology shift over the years, which is why the data are inhomogeneous, from subclinical Cushing syndrome to autonomous cortisol secretion (ACS), from expanding the concepts of “adrenal tumour” and “adrenal hyperplasia” to the new ones brought by the 2022 WHO (World Health Organization) classification, all of these being expected to increase the level of awareness among medical community, including reports on Connshing syndrome [12,20].

This recent classification introduces categories like sporadic nodular adrenocortical disease (for a single gland lesion) and bilateral micronodular/macronodular adrenal cortical disease (for bilateral adrenal hyperplasia), regardless of their hormonal profile which is important to recognise since both categories may be associated with Connshing syndrome [20].

The term “Connshing syndrome” itself is not used in most of the papers considered, other terminologies instead being used to describe it, and we accessed the topic through the combination of key words previously mentioned, which made the research more difficult. That is why we encourage the use of a single term to help increase awareness among clinicians. Some previous terminology is now inadequate due to recent progress, for example, “aldosterone/cortisol producing adenoma” does not comprise the entire category of Connshing syndrome, since PA may underline a bilateral adrenal involvement in addition to ACS, or a cortisol-producing nodule might be CYPB12 (aldosterone synthase) negative and be surrounded by aldosterone producing micronodules [27]. Due to the novel and continuously changing terms over the years, we will use both new and historic ones, in order to reproduce the clarity of the original studies and their included criteria. 

Of note, the term “Connshing syndrome” should not be extended to two other specific adrenal conditions: first, an adrenal cortical carcinoma which typically involves a challenging cocktail of different adrenal hormones, mostly cortisol, but also aldosterone and androgens; and, second, one characterized by the synchronous diagnosis of an aldosteronoma and a contra-lateral cortisol producing adrenocortical adenoma [28,29,30,31]. For an example of such a situation which does not fit into the Connshing group, we mention one case report published by the CONPASS group in 2020 of a 54-year-old male who was admitted for PA and subclinical Cushing syndrome, and presented synchronous detection of bilateral adrenal tumours: a right adrenal adenoma with positive CYP11B2 immunostaining (aldosteronoma) underlying a somatic *KCNJ5* mutation (Leu168Arg), and a left adrenal tumour with positive immunostaining for CYP11B1 (negative for *KCNJ5*), responsible for persistent hypercortisolism [31].

### 3.2. Epidemiological Aspects 

In essence, the prevalence of concurrent hypercortisolism in PA is higher than initially reported (going from “rare” to approximately 4–10% one decade ago) despite this entity not being designated with a distinct name in almost all clinical studies [32,33,34,35,36].

In recent times, autonomous cortisol and aldosterone producing tumours have required not only a hormonal confirmation, but also an immunohistochemistry report (as suggested by modern pathological approach of PA according to 2022 WHO), and, additionally, a molecular genetic workup in selected cases [20,37,38,39,40].

The individual scenario of Connshing syndrome detection may start from any of the indications to test Conn or Cushing syndrome, such as uncontrolled/resistant HBP, accidental detection of an adrenal neoplasia by performing different abdominal imaging procedures (adrenal incidentaloma), hormonal assays for patients with HBP and diabetes mellitus, osteoporotic fractures, evaluation for newly detected hipercortisolemia, hyperaldosteronism, etc. [35,37,38,39,41,42,43].

Connshing syndrome involves a combination of aldosteronoma or PA-related bilateral adrenal disease with mild persistent hypercortisolism, but the criteria of defining cortisol excess may vary, thus the overall prevalence [1,2,3,4]. In the German Conn’s Registry, 77.6% (*n* = 125) of 161 patients with PA had ACS. This is probably the highest ratio of ACS because it uses using one out of three types of assessments: either 1 mg dexamethasone (DXM) suppression test; late-night salivary cortisol (LSC); or 24 h urinary free cortisol (UFC) [44,45,46,47]. However, most data sustains a prevalence of 20–30% among PA patients (typically through a single evaluation, mostly 1 mg DXM inhibition test with a second day cortisol cut-off above 1.8 µg/dL) with the majority of (but not all) cases clinically manifested as subclinical Cushing syndrome, not overt Cushing syndrome [26,30,35,48].

### 3.3. Clinical Presentation: Cardiovascular Risk in Patients with PA and ACS

Cortisol excess in PA is a supplementary contributor to myocardial remodelling in PA. Aldosterone excess causes myocardial toxicity via anomalies of ion homeostasis, intracellular vacuolization and interstitial oedema; persistent hypercortisolemia induces cardiomyocytes hypertrophy and myofibrillolysis [49].

PA patients had left ventricular hypertrophy more often than subjects with essential arterial hypertension due to hormone excess effects on myocardium [50]. A study of 73 patients with Connshing syndrome (German Conn’s Registry) showed that 85% of them had left ventricular hypertrophy according to left ventricular mass index which correlated with total glucocorticoid excess (*p* = 0.018) and tetrahydroaldosterone excretion (*p* = 0.024), and significantly improved after adrenalectomy (*p* < 0.0001) in 45/73 cases with aldosteronoma, displaying a smaller effect under therapy with mineralocorticoid receptor antagonists (*p* = 0.024) in 28/73 subjects with bilateral adrenal hyperplasia. Multivariate regression confirmed the same correlation with cardiac hypertrophy only for glucocorticoids excretion [44]. This demonstrates high cortisol-associated cardiac damage in PA. 

ACS supplementary hits the vascular remodelling in PA individuals [51,52]. We mention here a study of 436 subjects with PA; 23% of them represented the ACS group (defined as a cortisol value more than 1.8 μg/dL after 1 mg DXM inhibition test) and they not only were associated with a higher prevalence of diabetes mellitus and an increased aldosterone/renin ration, but also had higher brachial-ankle pulse wave velocity versus the non-ACS group (*p* = 0.01), as well as a larger area of vascular fibrosis (*p* = 0.02). Both groups significantly improved this last mentioned parameter after one year of targeted therapy [53].

PA patients are at higher risk for renal damage when compared to individuals with essential arterial hypertension, especially if a patient is diabetic [54,55,56]. The co-presence of ACS aggravates the renal dysfunction [57,58]. One retrospective transversal study from 2022 on 1310 PA subjects identified a subgroup (*n* = 340) with Connshing syndrome (serum cortisol levels higher than 1.8 µg/dL after DXM suppression test) that associated a prevalence of low estimated glomerular filtration rate and proteinuria twice higher than non-ACS group, the presence of ACS being an independent risk factor to both parameters meant to describe the kidney damage [57]. A case-control study of 57 PA patients without ACS versus 57 subjects with ACS (with a value of plasma morning cortisol ≥1.8 μg/dL after DXM test) showed a higher prevalence of HBP (100% versus 52.7%, *p* < 0.0001) and lower glycated haemoglobin A1c levels (*p* = 0.028). Longitudinal components of the study pointed out that, after a median of 2.25 years, PA + ACS patients presented a statistically significant increase in renal dysfunction [58].

### 3.4. Glucose Profile in Patients with Connshing Syndrome 

Aldosterone excess might impair pancreatic insulin secretion and insulin sensitivity, while therapy in PA patients improves insulin response to glucose (which increases) and insulin clearance (which decreases) [59,60].

**Figure 1:**
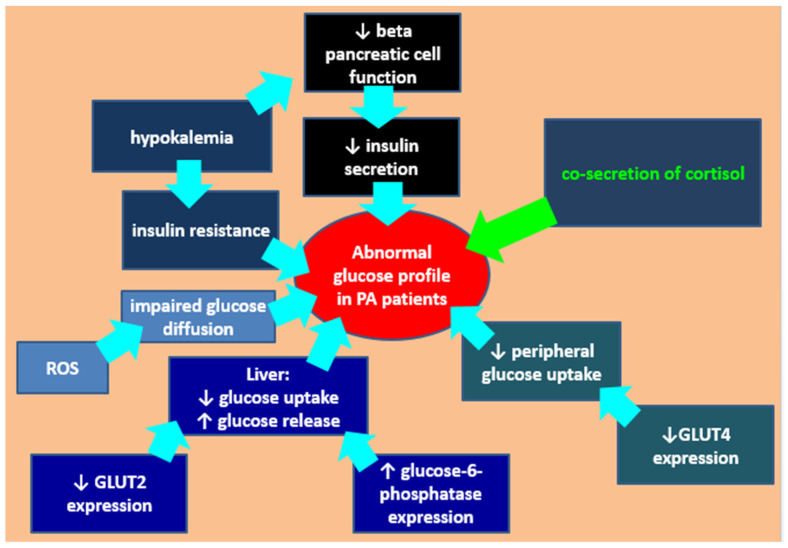
Mechanisms of glucose profile anomalies in patients with Connshing syndrome. In PA patients, apart from cortisol co-secretion, hypokalemia decreases pancreatic release of insulin, and insulin sensitivity in association with reduced glucose uptake by peripheral tissues (liver, skeletal muscle, adipose tissue); aldosterone-induced reactive oxygen species causing endothelial dysfunction impairs glucose diffusion; the decrease of beta pancreatic cell function causes a reduced insulin release; the reduced expression of glucose transporters translates into reduced hepatic uptake of glucose (via GLUT2), with low glucose uptake by other non-hepatic tissues due to reduced GLUT4 expression (please see [59,60,61,62]). Abbreviations: PA = primary aldosteronism; ROS = reactive oxygen species; GLUT = glucose transporter.

In PA patients, apart from potential cortisol co-secretion, hypokalemia decreases pancreatic insulin release and insulin sensitivity in association with reduced glucose uptake by peripheral tissues (for instance, liver, skeletal muscle, adipose tissue), while aldosterone-induced reactive oxygen species caused endothelial dysfunction and impaired glucose diffusion, all of which are contributors to glucose profile anomalies [59,60,61,62] (Figure 1). 

Aldosterone and cortisol excess are related to a higher rate of metabolic syndrome in adrenal tumours [60,61]. Mineralocorticoid receptors play an important role in blood pressure control, but also in metabolic components, being expressed in adipose tissue with potential activation by glucocorticoids, mechanisms that play an important role in PA and PA with ACS [62].

The German Conn’s Registry identified type 2 diabetes mellitus more often (by performing oral glucose tolerance test in each subject with PA) in patients with PA and ACS (*n* = 125/161) versus PA (20% versus 0.8%, *p* < 0.0001), and versus a population-based control group from the KORA study (20.6% versus 5.9%, *p* = 0.022), suggesting the additive influence of cortisol on glucose profile in subjects with PA, thus proving an index of suspicion concerning Connshing syndrome in PA individuals [45].

Additionally, a large multicentre cohort study (Japan Primary Aldosteronism Study Group) of 2210 PA patients identified a rate of diabetes mellitus—defined by a level of glycated haemoglobin A1c of 6.5% or above of 21.6%—higher than the general reference population (12%) with a significant contribution of ACS, attributed by a cortisol level of at least 1.8 µg/dL after a 1 mg DXM test [63]. A Chinese study of 555 PA patients identified 22 of them with Connshing syndrome, a group that showed a larger tumour diameter than the pure PA group (*p* < 0.05), but also had a higher ratio of cardiovascular complications and different types of glucose profile anomalies, as well as osteopenia and osteoporosis (*p* < 0.001), whilst 17/22 individuals harboured *KCNJ5* mutations [64].

### 3.5. Quality of Life and Neuro-Psychological Impairment in PA and Cortisol Co-Secretion

Cortisol excess contributes to impaired quality of life in PA sufferers through multiple co-morbidities, including depression/anxiety [65,66,67,68,69,70].

A German Conn’s Registry-based study showed that subjects whose PA was treated had improved anxiety and depression scores (based on self-reported questionnaires; *p* < 0.001), as well as an improved QoL mental score (*p* = 0.021) and physical score (*p* = 0.015), an aspect that was confirmed in the Connshing group. Depression scores improvement for males as well as females was statistically significant higher in this subgroup (*p* = 0.004, respective *p* = 0.011) when compared to those without ACS. Similarly, physical QoL score improved in females (*p* = 0.023) and the mental sub-score improved in males (*p* = 0.027) with ACS as opposed to non-ACS. More pronounced post-adrenalectomy effects on anxiety and depression in those who initially experienced cortisol excess are probably related to the neuro-psychical effects of glucocorticoids [46].

The role of aldosterone and/or cortisol activation in the pathophysiology of depression and anxiety cannot be described through a simple linear perspective. We already know that aldosterone, by binding to mineralocorticoid receptors, activates different mechanistic pathways for anxiety and depression; a reduction of its levels as seen in post-adrenalectomy status improves the aldosterone exposure of central nervous system areas, while mineralocorticoid receptors antagonists for PA patients target receptors from different locations, and some exposure to cortisol becomes the main ligand in persons with Connshing syndrome [71].

### 3.6. Bone Status in Relationship with Aldosterone and Cortisol over Production

PA is a contributor to osteoporotic fractures, especially vertebral fractures, independently of potential variations of HBP as a risk factor for fall. Parathormone-aldosterone interplay should be taken into consideration as well, including tertiary hyperparathyroidism as a complication of HBP and diabetes mellitus-induced end stage renal failure [72,73,74,75,76].

It is already known that seniors with a long history of hormonally active adrenal cortical tumours have a higher prevalence of osteoporosis, noting the well-known effects of glucocorticoids on bone in patients with Cushing syndrome and even ACS [77,78,79]. In PA patients, aldosterone excess contributes to bone loss and increased fracture risk, but also diabetes mellitus, ACS, and, probably, HBP and dyslipidaemia [73,74,75,80]. Concerning PA-ACS group analysis of bone loss and fracture risk, the volume of statistical evidence remains low. Further studies are necessary, starting from this theoretical. 

One retrospective cross-sectional study on 113 PA patients showed that unilateral PA is an independent risk factor for vertebral fractures. PA subjects had a higher prevalence of vertebral fractures (29% versus 12%, *p* = 0.011) versus non-PA subjects (*n* = 58). Unilateral PA (*n* = 37) versus bilateral PA (*n* = 76) had an increased risk of similar fractures (46% versus 20%, *p* = 0.021) [81]. One longitudinal observational study of 36 menopausal women with PA showed that PA patients had higher bone formation osteocalcin versus age and body mass index-matched controls (*n* = 18, *p* = 0.013), while the other bone turnover markers were similar. After 1 year of therapy with mineralocorticoid receptor antagonist spironolactone in 18 cases with bilateral PA, bone formation markers osteocalcin, P1NP (procollagen 1 N-terminal propeptide) and alkaline phosphatase were statistically significantly decreased versus the baseline. Patients who underwent unilateral adrenalectomy for aldosteronoma had similar values. This means that bone turnover might be affected in cases with aldosterone excess which are treated conservatively [82].

### 3.7. Biochemistry and Hormonal Workup

Screening tests for PA should be done in patients with very high values of arterial hypertension (ideally before starting anti-hypertensive drugs), resistant HBP, HBP and hypokalaemia, adrenal incidentaloma, as well as individuals who had a family history of HBP or strokes at young ages (before 40 years old) or PA [83,84,85,86,87,88]. After identification of a high aldosterone/renin ratio, confirmatory tests (saline infusion test or captopril test) may follow the screening assays, but not necessarily, unless spontaneous or diuretic-induced severe hypokalaemia or highly suggestive plasma aldosterone levels with suppressed renin values are presented and highly suggestive for the diagnostic [83,84,85,86]. Additionally, an ACTH stimulation test might distinguish between aldosteronoma and a bilateral PA disease, but not all studies agree [89].

The screening evaluation of cortisol excess typically requires a 1 mg DXM suppression test, or alternatively LSC and UFC, as for instance used in the German Conn’s Registry [44,45,46,47]. Rarely, the evaluation of cortisol circadian rhythm and DXM suppression test with higher doses (like 2 mg) are necessary [90]. As mentioned, steroids metabolomics represent the next step in approaching adrenal tumours, especially entities like Connshing syndrome [83,91].

PA is correlated with a pro-inflammatory status with secondary cardiovascular, endothelial, renal and skeletal effects, while cortisol co-secretion might be a contributor to opposite immunosuppressive effects, for instance on different human antibodies’ profiles [92,93,94]. A one year longitudinal study on 97 patients with PA from the German Conn’s Registry (74.2% with Connshing syndrome) showed a significant increase of anti-thyroperoxidase antibodies after adrenalectomy in patients with aldosteronomas who had at least one abnormal test concerning the diagnostic of ACS (*p* = 0.049), or at least two abnormal tests (*p* = 0.015), or an abnormal DXM inhibition test (*p* = 0.018). This was in contrast to there being no effect on thyroid antibodies levels in subjects with aldosteronoma without ACS or PA-related bilateral adrenal disease (*n* = 54), who were not referred for adrenalectomy [47].

### 3.8. Adrenal Venous Sampling

AVS is required in bilateral PA (including the ACS subgroup), and aldosterone as well as cortisol should be assessed; the procedure is not automatically available in many centres, and sometimes the results are inconclusive or display a high variability in PA, despite an experienced team [95,96,97,98,99,100,101,102,103]. However, AVS brings supplementary information to imaging procedures allowing better surgical decisions, with a success rate of 76–90%, and the presence of concomitant cortisol excess is mandatory for assessment [95,96,97,98,99,100,101,104,105].

Anti-hypertensive drugs like mineralocorticoids receptors antagonists should be stopped one month prior to AVS which is not feasible in severe HBP [106,107]. Not all studies agree that discontinuation of such medication as spironolactone impairs the lateralization index [107]. Since there are some technique differences between left-right AVS, recently an alternative to the aldosterone/cortisol ratio for right adrenal gland which associates a more difficult cannulation of right adrenal vein was suggested by the aldosterone/cortisol index between the left adrenal vein and the inferior cave vein [108,109,110].

Similarly, ACTH stimulation during AVC might provide some help, but it is not routinely indicated [111,112]. The rationale of this test (used in 40% of centres, but not with guideline indication) is to maximize cortisol secretion to avoid the daily pulsatile pattern [113,114]. Intra-procedural cortisol assessment during basal and ACTH-stimulated AVS might increase rates of success [115]. Liquid chromatography-mass spectrometry methods rather than immunoassays are useful for cortisol assays during AVS [116]. Clinical/(computed tomography) imaging-corrected indices for AVS might improve results, as well as rapid cortisol assays [117,118].

Cortisol co-secretion might lead to misinterpretation of non-ACTH (Adrenocorticotropic Hormone)-stimulated AVS. High cortisol (causing a decreased aldosterone/cortisol ratio) could falsely classify unilateral cases as bilateral [119,120]. Additionally, apart from PA, AVS might serve to distinguish between unilateral and bilateral ACS [121]. Another issue concerning glucocorticoid status and AVS in PA involves pre-medication with DXM in individuals with iodine contrast allergy potentially interfering with the aldosterone/cortisol ratio during AVS [122,123,124]. 

One retrospective case-control study of 144 adults with PA showed abnormal DXM suppression tests in 14.6% of them; the ACS group had an increased cortisol value at the level of inferior vein cave versus the non-ACS group (*p* = 0.01), but the selectivity and lateralization index and adrenal vein cannulation rate were similar among ACS andnon-ACS patients [125]. A retrospective, single centric, cohort study (from 2022) of 278 subjects with PA who were referred for AVS showed that 18.9% of them had an abnormal 1 mg DXM suppression test (post-DXM cortisol levels between 1.9 and 5 µg/dL—15.8% of them, with more than 5 µg/dL—another 2.9%); lateralized cases from the second group had a lower lateralization index versus those with post-DXM cortisol levels below 5 µg/dL, suggesting that high cortisol may impair the accuracy of the left-right gradient [126].

### 3.9. Advances in Molecular Pathology and Genetics

Adrenal tumours display a highly heterogeneous profile with respect to molecular and pathological/immunohistochemistry profile, as well as genetic background, and this field has recently seen spectacular progress [12,13,14,15,16,17,18,19,20,127,128,129,130,131,132,133,134].

Traditionally, PA is associated with an adrenal tumour, bilateral adrenal hyperplasia, adrenal cortex carcinoma and ectopic aldosterone production, but benign adrenal sources are described as aldosteronona (solitary tumour), aldosterone-producing nodule or multiple micronodules (non-classical type), respective bilateral adrenal disease; for instance, unilateral PA may underline a unilateral single or multiple aldosterone-producing adenoma or an aldosterone-producing cell cluster [12,13,14,15,16,17,18,19,20,127,128,129,130,131,135,136].

PA is caused by somatic mutations of *KCNJ5* (with predictive potential), *CACNA1D*, *CACNA1H, CLCN2, ATP1A1* and *ATP2B3* genes which are found in 60–80% of sporadic aldosteronomas (mostly *KCNJ5*). They involve ion channels or pumps associated transmembrane exchange triggering calcium-related signal transducing pathways that leads to aldosterone overproduction [127,128,129,130,131,137]. Familial PA (bilateral glands involvement in familial PA type I, II, III and IV) is associated with germline mutations of *KCNJ5, CACNA1H* and *CLCN2* genes, and probably *PDE2A* and *PDE3B* variants [128,129,130,131]. Recently, CYP11B2 staining in addition to a genetic workup improved the characterization of PA. The 2022 WHO classification recommended the routine use of HISTALDO (histopathology of PA) classification to describe CYP11B2 immunohistochemistry ofdifferent areas of adrenal lesions with potential aldosterone production [20,130,131].

On the other hand, non-cancer-related Cushing syndrome involves positive adenomas for various somatic mutations of *PRKACA, PRKAR1A, PRKACB*, *GNAS, PDE11A* and *PDE8B* (cyclic AMP signalling pathways) genes and primary bilateral macronodular adrenal hyperplasia (PBMAH) underlying inactivating mutations of thegermline *ARMC5* gene (20–25% of all PBMAH cases), menin, *GNAS*, as well as the somatic *KDM1A* gene (identified in >90% of PBMAH with food-dependent Cushing syndrome) [138,139,140,141,142].

Thus, we believe that a shift concerning genetic approach of unilateral adrenal tumours has been made, a genetic workup no longer being relevant only in bilateral disease [143,144]. Unilateral and bilateral lesions seem distinct players in the same field of hormonally active adrenal tumours; similarly, we might say that ACS appears as an additional player in the PA field, regarding both aldosteronoma and bilateral adrenal hyperplasia. In situ metabolomics is correlated with a CYP11B1/B2 stain and tumour genotype [145,146]. Aldosterone synthase (CYP11B2)/cortisol synthase (CYP11B1) immunostaining profiles represent a hallmark of the modern approach regarding unilateral tumours with aldosterone and/or cortisol excess. CYP11B2/CYP11B1 profiles correlate with clinical presentation and outcome [147,148].

One retrospective study from TAIPAI registry (a 2022 paper) showed that 36.7% of patients with PA and ACS (*n* = 98) had mutations which are related to cortisol producing adenomas (*PRKACA* and *GNAS*), and this subgroup of Connshing syndrome associates a higher value of cortisol after a 1 mg DXM inhibition test (5.6 μg/dL) when compared to the subgroup without these mutations (2.6 μg/dL, *p* = 0.003), as well as a larger diameter (2.22.2 ± 0.3 versus 1.9 ± 0.7 cm, *p* = 0.025). There is a similar post-adrenalectomy outcome in both sub-groups [149].

One histological-based retrospective study from 2022 on 56 nodules from 24 individuals with unilateral PA showed that 30.4% of nodules had *KCNJ5* mutations (all CYP11B2 staining positive), but CYP11B2/CYP11B1 expression was heterogeneous as pattern and intensity in both, this *KCNJ5* positive group, as well as the *KCNJ5*-Wild Type category [150]. Another study of 61 unilateral cases of PA showed that 65.6% of them had aldosterone producing nodules/micronodules, whilst ACS was confirmed in 32% of the entire cohort which correlated with a larger tumour (>1.98 cm) [151]. Bilateral adrenal disease also relates to aldosterone and cortisol excess. One study from 2022 of 777 patients (495/777 were males) with bilateral adrenal tumours showed that PA and PBMAH are the most frequent hormonally active lesions (16.6% with PA, and 8.8% with PBMAH) [152].

### 3.10. Therapy & Outcome in Connshing Syndrome

In PA and subclinical or clinical Cushing syndrome, the goals of treatment are to normalize both blood pressure and excessive aldosterone/cortisol production, to reduce associated comorbidities, to improve the quality of life and to reduce disease-related mortality [153,154,155,156,157,158,159].

Bilateral PA is treated with mineralocorticoid receptor antagonists, if a unilateral source of aldosterone and/or cortisol source is not indicated by AVS to be a surgery candidate. Laparoscopic adrenalectomy, typically with a good outcome and a significant post-surgery remission/improvement of the clinical picture, remains the treatment of choice in unilateral PA and patients with Cushing syndrome, thus (indirectly) also in patients with Connshing syndrome which is not the subject of any distinct guideline/protocol, though it is included as a particular section in recommendations for PA patients [153,155,156,157,158,160,161].

Post-operative short and long term outcomes depend on several factors, including age, sex, body mass index, genetic background, pre-operative duration of the adrenal disease, adequate use of AVS, optimal choice of imaging techniques and the presence of ACS in PA patients [154,162,163,164,165,166].

Suppression of aldosterone at the level of the contralateral adrenal gland in aldosteronoma, as reflected by AVS, predicts a good post-operatory outcome [167]. One prospective study on 82 aldosteronoma patients who were referred for laparoscopic adrenalectomy showed that pre-operatory plasma cortisol higher than 1.8 μg/dL after a 1 mg DXM test was identified in 22% of the subjects (ACS group). Patients with post-DXM levels above 1.5 μg/dL had larger tumours than below this cut-off (*p* = 0.02). *KCNJ5* mutations and body mass index inversely correlated with post-DXM cortisol >1.5 μg/dL. Individuals with positive immunostaining for CYP11B1 correlated with wild-type *KCNJ5* (*p* = 0.018), respective CYP11B2 positive was more often found in tumours with *KCNJ*5 mutation (*p* = 0.007). A lower rate of clinical remission after surgery was identified in subjects with post-DXM cortisol >1.5 μg/d and wild-type *KCNJ5* (36.8%). Thus, Connshing syndrome seems more frequent in aldosteronomas with negative *KCNJ5* and larger tumours, being correlated with a reduced rate of clinical success (36.8%) [168].

Theoretically, there is a higher risk of developing post-adrenalectomy adrenal insufficiency in Connshing syndrome than PA patients, but not all studies agree [169]. However, since this entity is not routinely pre-operatively assessed as a distinct subtype of PA, the post-surgery cortisol assays (baseline, but also and most importantly, dynamic stimulation tests) might not always be available. The use of Cosyntropin (synthetic ACTH) stimulation testing during the second day after surgery identifies the patients with adrenal insufficiency due to prior glucocorticoids excess. 

One prospective on patients with adrenal cortex tumours referred to unilateral adrenalectomy identified that half of the 12 patients with Connshing syndrome had with post-operatory adrenal insufficiency, specifically a very mild form requiring glucocorticoid replacement for a median of 0.8 months as opposed to those with pre-surgery overt Cushing syndrome (requiring glucocorticoids for a median of 6 months), and to those with mild ACS (a median of 2.1 months for substitution) [170].

Synacyhen (Cosyntropin) stimulation testing should follow unilateral adrenalectomy in patients who presented an abnormal pre-operatory DXM test (mostly subclinical), unless there is clear evidence of adrenal insufficiency which is very rare in Connshing syndrome. One study of a small size showed that more than 56% of the patients with subclinical Cushing syndrome had an optimal cortisol response during a short/rapid test of Cortrosyn, and none developed clinically manifested adrenal insufficiency. The identification of adequate cortisol response to stress might avoid unnecessary post-operative glucocorticoids exposure [171]. The presence of ACS in PA patients, which correlates with lower insulin during the oral glucose tolerance test and inadequate suppression during the DXM test, is associated with a suboptimal response to ACTH stimulated cortisol after unilateral adrenalectomy [172].

## 4. Discussion

### 4.1. Sneak Peek to Connshing Syndrome

The concept of Connshing syndrome has a long standing scientific history, despite not being recognized under a dedicated nomenclature [1,173,174,175]. It is mainly defined alongside PA rather than Cushing’s syndrome. However, the current progress in this particular area has dramatically reshaped the traditional models, requiring us to quote a paper of Dr. N.T. Brown from 2011 entitled “This is not Dr. Conn’s aldosterone anymore” [176]. Both Conn syndrome and Cushing syndrome belong to the group of more than 15 causes of endocrine hypertension, explaining their importance in daily practice [177]. Connshing syndrome seems like an overlapping entity, but further expansion of translational molecular genetics will prove if, in fact, this is a distinct condition, as we are inclined to believe based on current evidence. In endocrinology we now recognize previously overlapping entities with a distinctive description, as shown by the 2022 WHO classification, seen for instance in pituitary neuroendocrine tumours (PitNET) with different immunostaining and pituitary transcription factors profiles [178].

**Figure 2:**
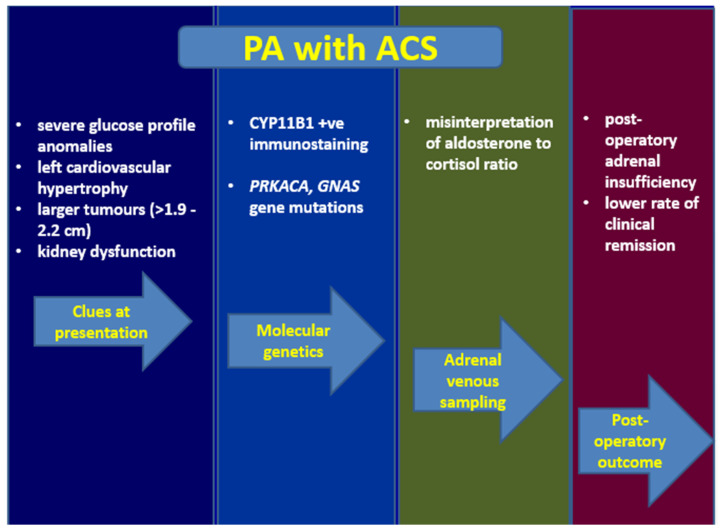
Overview of Connshing syndrome (primary hyperaldosteronim and autonomous cortisol secretion), from index of suspicion to post-adrenalectomy outcome (see references within the main text). Abbreviations: PA = primary aldosteronism; ACS = autonomous cortisol secretion; AVS = adrenal venous sampling.

In PA patients, we should suspect cortisol co-secretion in cases with a more severe glucose and cardiovascular profile, left ventricular hypertrophy, larger tumours than 1.9–2.2 cm, and, of course, in extremely rare cases with typical Cushing syndrome and PA (outside of a diagnosis of an adrenal cortical carcinoma); additionally, ACS seems to be an independent risk factor for renal damage in PA, and probably also for depression/anxiety and osteoporotic fractures, despite a lower level of statistical evidence [35,48,57,179] (Figure 2).

Approximately one-third of the PA-ACS group harbour mutations of cortisol producing lines like *PRKACA* and *GNAS* with higher cortisol levels and tumour diameter than PA-ACS subjects without these mutations [149]. The aldosterone-producing tumours and ACS have a higher rate of not harbouring *KCN*J5, but *KCNJ5* gene mutations have been described in ACS-PA, too [180,181]. Pitfalls of hormonal assessments in Connshing syndrome involve the index of suspicion meaning seeking for ACS in PA subjects and the interpretation of aldosterone-to-cortisol ratio during AVS [182,183,184].

Aldosteronoma removal might be associated with a lower chance of complete clinical remission in cases with ACS, but cortisol levels after surgery significantly decrease [174]. No particular aspects are mentioned surrounding the surgical procedure itself, with a standard recommendation for laparoscopic adrenalectomy [185,186,187]. Nevertheless, postponing surgery and/or long-term therapy with mineralocorticoids receptors antagonists may not act effectively against excessive amounts of cortisol, thus the long term prognosis in such cases should be re-considered [188].

Development of clinically manifested adrenal insufficiency is rare, thus a Cortrosyn stimulation test might be helpful to avoid unnecessary glucocorticoids drugs as post-operative medication. If a stimulation test is not available, follow-up of blood pressure levels or event intermittent glucocorticoids replacement under stress circumstances (like infections or intense physical effort) are useful, since corticoids exposure may be necessary only occasionally due to partial adrenal insufficiency [189]. Prior studies reported adrenal insufficiency after adrenalectomy for aldosteronoma, but it is likely that cases with ACS were not analysed before surgery. For example, one prospective study on PA sufferers who were referred for adrenalectomy identified an incidence rate of 6.9 adrenal crises per 100 patient-years during a cumulative surveillance of 14.5 years [190].

### 4.2. Current Limits 

Despite various terms describing the mixed condition, a large amount of quality data have been published up until the present time, which have became even more spectacular within recent years due to molecular genomics and immunohistochemistry for the CYP11 system, replacing the traditional system of simple pathological report.

Ongoing data on translational genomics need further implementation into daily practice. Many countries do not have a reimbursement protocol of immunohistochemistry profile and gene testing in adrenal tumours, while AVS is not available in many endocrine centres. There are still unknown areas concerning routine use of molecular and genetic assessments. For instance, we mention an adult female case who was diagnosed with Connshing syndrome followed by the detection of ovarian hyperthecosis-associated hyperandrogenemia. Whether a particular genetic configuration links ovarian tumours with Connshing syndrome is still on open issue [173].

We do not have enough evidence on longitudinal studies concerning the evolution of Connshing syndrome under different medical regimes as opposed to PA and subclinical Cushing syndrome. 

Additionally, when it comes to bone effects of PA and ACS, there is an open discussion which method of fracture risk assessment should be indicated, and which is the best order of the selection of patients to be assessed. So far, bone mineral density is best provided by the gold standard DXA (Dual Energy X-Ray Absorptiometry) in postmenopausal women and male seniors [74,78,191,192,193,194,195]. Trabecular Bone Score is particularly useful in menopausal diabetic females and glucocorticoid excess, according to general recommendations [196,197]. There is also the issue of when to perform a screening (plane) X-Ray profile of the thoracic and lumbar spine in patients with PA, especially the Connshing group, noting that the vertebra is the most frequent site of fracture identified in these categories [198].

## 5. Conclusions

The importance of recognizing that some adrenal lesions might bring together the entire multidisciplinary panel of both Conn and Cushing syndromes starts with awareness and continues to early diagnosis, hormonal assays including AVS, molecular genetics and life time management. Promoting it as a distinct entity in the growing statistical evidence on this particular subject provides better knowledge, and a step forward towards finding an optimal approach. Connshing syndrome should be considered distinctly in new clinical and pathological classifications of PA to gain more attention from the medical community. 

## Abbreviation

ACTH	Adrenocorticotropic Hormone
ACS	autonomous cortisol secretion
AVS	adrenal venous sampling
DXM	dexamethasone
HBP	high blood pressure
PA	primary aldosteronism
PBMAH	primary bilateral macronodular adrenal hyperplasia
P1NP	procollagen 1 N-terminal propeptide
PitNET	pituitary neuroendocrine tumors
WHO	World Health Organization
LSC	late-night salivary cortisol
UFC	24-h urinary free cortisol
